# Eliminating trachoma: accelerating towards 2020

**Published:** 2016

With less than four years to reach the elimination target for trachoma, we need to tackle this preventable disease head-on. Through the powerful unity of the Alliance for GET2020, there is global commitment to eliminating a disease that has existed for thousands of years.

According to data released in April, around 200 million people are at risk of trachoma, 1.2 million people are blind and 3.6 million need surgery to avoid blindness. Based on current estimates, an additional US$700–800 million is needed to implement the SAFE strategy and eliminate trachoma globally by 2020.

Thanks to recent progress resulting from intensive efforts by partners, we now have an accurate understanding of where trachoma exists, how to treat it, and at what cost – and we have the antibiotics necessary to do so.

**Figure:**
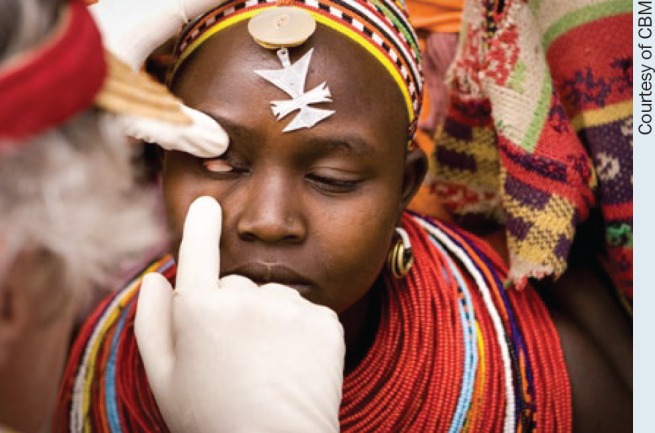
8-year-old Namaria has her eyes checked for trachoma. KENYA

Whilst there are many successes to celebrate in the concerted efforts of the past five years, the trachoma community recognises the challenges in achieving global elimination targets: increasing coverage, identifying transmission routes, engaging other sectors for sustainability, and attracting the funding needed for elimination efforts in the 43 countries requiring SAFE interventions.

*Eliminating Trachoma: Accelerating Towards 2020* launched as an online publication in June 2016. Targeting funders, policy makers and implementing partners, the publication outlines the current disease burden, defines elimination challenges and priorities and communicates a strong call to action for continued and increased support of trachoma elimination.

Visit **www.trachomacoalition.org** for more information.

