# Supplementary material for: Eliminating trachoma: accelerating towards 2020
Source: Community Eye Health. 2016;29(93):18. (PMC4995842)
Supplement: Related texts

# Brief report: Free online course for trachoma programme teams

**Published:** 2016

**Discover how communities and experts are joining together to end trachoma across 51 endemic countries by the year 2020.**

200 million people are affected by trachoma. It's a slow and painful way to go blind. But we now have the tools and knowledge to confidently plan for its elimination as a public health problem by the year 2020.

We have a global district-by-district map that is nearly 100% complete and an intervention strategy, known as the SAFE strategy, that is promoted to reduce infection (by the organism *Chlamydia trachomatis*) and the subsequent scarring of eyelids and in-turning of eyelashes that leads to blindness.

Trained teams from the health, education and environment sectors collaborate to plan and deliver SAFE activities using protocols agreed by the international trachoma alliance GET 2020. The SAFE strategy involves:

- Surgery to correct in-turned eye lashes
- Antibiotic to reduce chlamydia infection
- Face washing to promote facial cleanliness to reduce transmission
- Environment improvement through provision of adequate water and sanitation to reduce transmission.

A new, free, online course aims to support and inform the teams delivering trachoma control activities who are implementing a programme to eliminate trachoma blindness in their community.

Developed at the London School of Hygiene and Tropical Medicine by an international team of trachoma and SAFE strategy experts, the course focuses on the actions that need to take place at the local community level to eliminate trachoma. Over 5 weeks, participants on the course will cover essential topics, including:

- Mapping trachoma to identify the affected communities
- Implementing the SAFE strategy at the community level
- Validating and certifying successful trachoma elimination

Course participants will take part in a variety of engaging and interactive learning activities throughout the course, including:

- Animated presentations and expert talks
- Quizzes and optional interactive exercises
- Articles and discussions
- Real life case studies

Course materials are optimised for access in settings with irregular or low quality internet access. For example, videos are also made available as downloadable presentations in text and PDF formats. Materials are published as Open Educational Resources (OER) so they can be freely shared, adapted and re-used by learners and educators. They will remain available to all learners after the course completion.

The course is suitable for:

- Trachoma programme managers and teams
- Eye health workers in affected regions
- Those involved in improving water and sanitation provisions
- Those interested in neglected tropical diseases.

The course can be taken as an individual or as a group. If you work in a team we encourage you to all sign up and learn together on the course.

The course will offer a great opportunity to learn from the experiences of participants from many other countries fighting to end trachoma. More than 3,000 participants from 118 countries took part in the similar Global Blindness course on Futurelearn last year.

If you are already involved with, or simply interested in, action with communities to eliminate trachoma by 2020, we hope this course will inform and inspire you. Your participation can be critical to progress in the elimination of trachoma blindness.

The course will launch in the first week of September 2016. To find out more and register your interest in the course please visit **https://www.futurelearn.com/courses/eliminating-trachoma** or email **Romulo.Fabunan@LSHTM.ac.uk**
